# Predictors of Social Media Use in Two Family Generations

**DOI:** 10.3389/fsoc.2021.813765

**Published:** 2022-01-13

**Authors:** Kristiina Tammisalo, Mirkka Danielsbacka, Emilia Andersson, Antti O. Tanskanen

**Affiliations:** ^1^ Department of Social Research, University of Turku, Turku, Finland; ^2^ Population Research Institute, Helsinki, Finland

**Keywords:** intergenerational relations, kin networks, social media, online, parent-child dyad

## Abstract

Older adults have recently begun to adopt social media in increasing numbers. Even so, little is known about the factors influencing older adults’ social media adoption. Here, we identify factors that predict the use of social media among older adults (aged 68–73) and compare them to those of their adult children (aged 19–56) using population-based data from Finland. As predictors for social media use, we utilized demographic factors as well as characteristics of the respondents’ social lives. In addition, we test whether social media use in older adults is predicted by the social media use of their adult children. The data used in this study uniquely enable the study of this question because actual parent-child dyads are identifiable. In both generations, women and those with higher education were more likely to use social media. Predictors specific to men of the older generation were being divorced and younger, and predictors specific to women of the older generation were having better health and more frequent contact with friends. A higher number of children predicted use in both men and women in the older generation. As for the younger generation, specific predictors for social media use in women were younger age, divorce, higher number of children, and more frequent contact with friends. For men in the younger generation, there were no significant predictors for social media use besides higher education, which predicted social media use in all groups. Finally, social media use in a parent representing the older generation was predicted by the social media use of their adult children. This study provides novel information on the predictors of the use of social media in two family generations.

## Introduction

The use of social media has become increasingly widespread among older adults. For instance, according to United States statistics, over 54-year-olds formed the fastest growing group of social media users in 2019, reaching adoption rates of 59% among 55–73-year-olds and 28% among over 74-year-olds (Vogels, 2019). Similarly, in Finland, approximately half of 55–74-year-olds use social media (Statistics Finland, 2020), and roughly one in four in this age group say that they check their social media incessantly or many times a day (Hanifi, 2019).

Although Finland can be considered a forerunner in digitalization, the use of digital tools among older adults is highly divided. While nearly half of older adults have adopted social media, the other half remain non-users. Such disparities in Internet use are referred to as *second-level digital divides* (e.g., Büchi et al., 2016). The first-level digital divide implies disparities in access to the Internet, whereas the second-level digital divide separates different types of use, such as whether one uses social media or not. With this study we aim to provide answers to the questions: How do the two sides of this divide, namely social media users and non-users, differ, and whether the differentiating factors vary depending on generation.

Some studies have found that being younger, female, more highly educated, and employed are at least partially accountable for higher social media adoption rates (Bell et al., 2013; Richter et al., 2013; Hutto et al., 2015; Vošner et al., 2016; Yu et al., 2016; Anderson and Perrin, 2017; Newman et al., 2019). However, other studies have not replicated these findings in samples of older adults. For example, a study from Taiwan on over 60-year-olds did not find age, gender, education, or employment status to be associated with social media use (Yu, 2020). Similarly, a United States study found no difference in income, wealth, or education between social media users and non-users over the age of 50 (Yu et al., 2016). These studies also included health and marital status as predictors but did not find correlations between them and social media use. Finally, a study of older Europeans suggests that education may, in some cases, be inversely related to social media use—those with less education use social media more rather than less (Richter et al., 2013).

Some of these mixed findings could be attributable to differences in the age range of the studied populations. The relative weight of the determinants behind social media use and non-use most likely shift depending on age and life stage (Yu et al., 2016). A clear example of a factor influencing social media use more among older adults than among younger adults is competence with technology (e.g., Friemel, 2016; Lüders and Brandtzæg, 2017). As for younger adults, socio-economic status may be a predictor of social media use (e.g., Baumer, 2018) which then loses its significance among older adults.

An especially salient factor influencing older adults’ social media use, and one of our main interests in this study, is the social influence from the part of friends and family (e.g. Friemel, 2016; Yu, 2020). It is well documented that social media are used for different purposes among younger versus older adults. These differences in use purposes place distinct selective pressures depending on life stage: The emphasis of social media use in younger adults is in the maintenance of networks of acquaintances and non-intimate friends (i.e., weak-ties networks), and this selects for users who are able to derive value, for example career opportunities, from such networks (e.g., Chan, 2015). For older adults’, on the other hand, social media provides an opportunity to reinforce strong ties, such as relationships with family members, selecting for users who are able to derive value, and well-being from social media engagement within such relationships (Zickuhr and Madden, 2012; Chan, 2015; see also; Hutto et al., 2015).

Correspondingly, non-use among younger versus older adults are differentially determined: Non-use among younger adults may be more strongly determined by labor market position (e.g., Baumer, 2018), whereas among older adults, those with few or no strong-tie relationships are less likely users and more at risk for digital exclusion. Notably, initiatives aiming at increasing technology adoption among older adults often place importance on the involvement of family members as a way to narrow the divide (e.g., Cornejo et al., 2013; Luijkx et al., 2015), while the lack of such strong ties remains unaddressed and a major factor behind non-use. Therefore, the current demographic trends of diminishing family sizes and population ageing are linked to digital divides and growing in importance.

Accumulating evidence shows that characteristics of social life may be major predictors of social media use in older adults (e.g., Nef et al., 2013; Friemel, 2016; Aarts, 2018; Hänninen et al., 2021), but this question has rarely been studied statistically with representative data. Furthermore, generational differences have not been systematically compared. Some studies indicate the kinds of characteristics of social life that may influence social media use among older adults: First, older adults who frequently meet with friends or family have been found to be more likely social media users (Richter et al., 2013). Second, even among older adults social media use is positively associated with the number of weak ties, that is, acquaintances and non-intimate friends, but it is not associated with the number of strong ties, that is, close friends and family (Yu, 2020). Third, as individuals age, the focus of social media use shifts from friends to family (Zickuhr and Madden, 2012; see also; Hutto et al., 2015), indicating that family members may be a motivator for social media use in older age.

Younger family members, in particular, seem to play an important role in older adults’ social media adoption (Nef et al., 2013; Aarts, 2018; Hänninen et al., 2021). It is well-known that involvement in the lives of children and grandchildren, for those who have them, is a significant source of vitality (Danielsbacka and Tanskanen, 2016). Therefore, it is no surprise that younger family members are often mentioned in interviews and polls as primary reasons for using social media (Zickuhr and Madden, 2012; Nef et al., 2013; Aarts, 2018; Newman et al., 2019; Yu, 2020). Moreover, children and grandchildren may actively encourage their parents and grandparents to adopt social media (Bell et al., 2013; Jung et al., 2017; Aarts, 2018; Yu, 2020), and they may provide the technical assistance to make it possible (Aarts, 2018; Hänninen et al., 2021). Thus, for many older adults, relationships with children and grandchildren may be a gateway to social media; conversely, those who do not have children or grandchildren, or whose children are not social media users, may be less likely to use social media.

It is plausible that social media adopters represent those with prior social advantages, that is, either larger social networks, more frequent social contact, and/or younger family members who are actively involved in the lives of their older relatives. It is also possible that social media further reinforces existing social advantages (e.g., van Deursen and Helsper, 2015). As a body of evidence indicates, social media may help older adults to stay socially active and feel less isolated (e.g., Chen and Schulz, 2016). It may also foster social satisfaction (Bell et al., 2013; Hutto et al., 2015), life satisfaction (Gaia et al., 2020), social inclusion (Richter et al., 2013), and boost social capital both online and offline (Nguyen et al., 2020; Simons et al., 2021).

So far, little is known about how family and friends influence social media adoption in older adults. In particular, the empirical evidence on the role of younger relatives influencing social media adoption of older adults is based on either qualitative or small and non-representative quantitative studies; therefore, the evidence may be considered merely preliminary.

In this study, we use population-based Finnish data to identify explanatory factors behind the use and non-use of social media in older adults and compare them to younger and middle-aged adults. As explanatory factors, we include demographic variables as well as characteristics of the respondents’ social lives (i.e., the number of close friends and relatives, as well as their frequency of contact with them). We examine how the demographic factors and the characteristics of the respondents’ social lives relate to the use and non-use of social media. We compare the two generations in order to better understand how the factors determining the use and non-use of social media vary depending on the generation. We also chart with whom respondents from each generation are in contact *via* social media. Finally, we investigate whether social media use in a parent representing the older generation is predicted by social media use in their children from the younger generation. The data used in this study uniquely enable the study of this question because actual parent-child dyads from the same families are identifiable.

## Materials and Methods

### Sample

The present study utilizes population-based survey data from the Generational Transmissions in Finland (Gentrans) project. The Gentrans data incorporate information on two family generations: the older generation born between 1945 and 1950 and their adult children born between 1962 and 1999. Data were collected by Statistics Finland in autumn 2018 and 2019 and comprise a nationally and geographically (excluding Åland) representative sample of 1,945 younger and middle-aged adults aged 19–56 years (mean = 42, SD = 5.86; younger generation) and 2,663 older adults aged 68–73 years (mean = 71, SD = 1.70; older generation). In addition, the data capture 1,003 family lineages and, therefore, enable integration of responses acquired from parents and their adult children who represent the older and younger generations, respectively.

The surveys were self-administered questionnaires which were posted to the respondents and returned also via post. The respondents were also given the option to access and submit the questionnaire online. The response rates were 66.4% for the older generation and 55.6% for the younger generation, of which 8.3 and 18.3%, respectively, were online submissions. Ethical permission for the first and subsequent Gentrans surveys were granted by the Ethical board of Statistics Finland in 2006. The users of the data have also committed to follow the Statistics Finland ethical rules by accepting The Pledge of Secrecy of Holder of Permission to Use Data. The respondents have given their informed consent to the use of their data for research, and the data have been anonymized.

### Variables

The dependent variable (i.e., social media use) is based on the question, “Do you use social media?” with the response options “yes” and “no.” An accompanying text read, “By social media we mean, for example, Facebook, Instagram, Twitter, and Snapchat.” Those who answered “yes” were additionally asked, “With whom do you interact via social media?” Response options were the following: mother, father, stepmother, stepfather, mother-in-law, father-in-law, daughter(s) (incl. step or foster), son(s) (incl. step or foster), son(s)-in-law, daughter(s)-in-law, grandchild (ren), sister(s), brother(s), niece(s)/nephew(s), aunt(s), uncle(s), cousin(s), friend(s), coworker(s), neighbor(s), and “someone else.” The latter question was used to characterize typical social media contact networks in the older and younger social media users, that is, to examine what types of family and non-family most typically are in the contact lists of younger and older social media users.

As independent variables, we utilized the following demographic variables. *Age* was calculated according to the year of birth. *Gender* was collected as a binary variable (male/female). *Education* was collected as a nine-level ordinal variable ranging from incomplete elementary school to doctorate degree. Subsequently, *education* was recoded as a four-level ordinal variable from 1 = completed or incomplete elementary school to 4 = higher education. Self-rated *economic situation* was measured on a four-level ordinal scale ranging from 1 = wealthy to 4 = low income. The scale was inverted for analysis. *Marital status* was collected as a categorical variable with six response options and subsequently recoded as the following categories: 1 = unmarried, 2 = cohabiting/married, 3 = divorced, and 4 = widowed. In the younger generation, divorced and widowed individuals were combined into one category because of the paucity of observations in the widowed category. *Health* was collected as an ordinal variable with a five-point scale ranging from 1 = very good to 5 = very poor. The scale was inverted for analysis so that higher numbers indicated better health.

In addition to the demographic variables, the independent variables also included characteristics of respondents’ social lives. These included the following variables: *number of close friends*, *number of children*, and *number of relatives* who are perceived as close but who do not live in the same household. In the variables *number of close friends* and *number of close relatives*, outlier values from 21 and up were set at 20. Missing values were replaced with mean values. Two additional variables characterizing the respondents’ social lives were *contact frequency with closest friend* and *contact frequency with closest relative during the past year* measured on a scale ranging from 0 = not once to 5 = several times a day. In the latter, the relatives given as options were: parents, siblings, nieces, and nephews and, for the older generation, also children and grandchildren. The relative with the highest value (i.e., with whom the respondent was most frequently in contact) was used in the analysis. For the older generation, children (mean 2.86) and mothers (mean 2.81) were the most contacted relatives. For the younger generation, mothers were the most frequently contacted relative (mean 3.06).

### Analytical Strategy

We used logistic regressions to test the extent to which the independent variables (listed previously in more detail) predict social media use separately in the two generations. We also tested the interaction effects of generation and gender to see whether the effects of the independent variables differ for the two generations or between genders. We then selected descriptive statistics regarding respondents’ social media contacts. These descriptive statistics show whether generations differ regarding with whom they most commonly interact via social media. Finally, we investigated whether social media use among respondents from the older generation is predicted by social media use in their adult children. This test was enabled by the Gentrans data, which capture information from related individuals, that is, from actual parent-child dyads representing the older and younger generations. To study this question, the data were reshaped into a long format so that the data included one parent and one adult child from the same family. Because the long format data were clustered within kin lineages, we used statistical software Stata’s (version 16.1) cluster option to compute the standard errors.

## Results

Social media use in the older generation was considerably less prevalent—42% compared to the younger generation’s 80% (*p* < 0.001). Descriptive statistics (Table 1) show the means and distributions of the independent variables.

**TABLE 1:** Descriptive statistics (*n* and %/mean).

	Older generation			Younger generation		
	*n*	%/mean	SD	*n*	%/mean	SD
Age (mean)	2,663	70.5	1.68	1,945	42.3	5.86
Gender (%)						
Women	1,566	58.8	-	1,252	64.4	-
Men	1,097	41.2	-	693	35.6	-
Education (%)						
Elementary school	783	30.3	-	50	2.6	-
Upper secondary/Vocational	1,244	48.1	-	731	37.8	-
College/Lower university degree	228	8.8	-	528	27.3	-
Higher academic degree	331	12.8	-	624	32.3	-
Economic situation^a^(mean)	2,624	1.8	0.79	1933	2.0	0.75
Marital status (%)						
Married/Cohabiting	1886	6.2	-	1,479	76.0	-
Unmarried	164	71.5	-	295	15.2	-
Divorced	321	12.2	-	165	8.5	-
Widowed	268	10.2	-	6	0.3	-
Health^b^(mean)	2,630	3.5	0.74	1,939	4.0	0.74
Number of friends (mean)	2,663	5.1	3.73	1,945	4.3	3.27
Number of close relatives (mean)	2,663	6.0	5.81	1,945	4.6	3.88
Number of children (mean)	2,663	2.1	1.52	1,945	1.8	1.47
Contact freq. with closest friend^c^ (mean)	2,382	2.3	1.19	1,888	3.1	1.20
Contact freq. with closest relative^c^ (mean)	2,663	3.2	1.09	1,945	3.3	0.99

a 1 = “low income,” 4 = “wealthy”.

b 1 = “very poor,” 5 = “very good”.

c 0 = “not once,” 5 = “several times a day”.

Logistic regression analysis revealed the associations between the independent variables and social media use. The regression coefficients are presented in Table 2 (older generation) and Table 3 (younger generation), with separate columns for men and women. Table 2 shows that age was negatively associated with social media use in the men of the older generation, meaning that older men were less likely to use social media than younger men, with predicted probabilities of being social media users dropping from 42% in 68-year-old men to 30% in 74-year-old men (see Supplementary Table S3 in Appendix). Negative association between age and social media use was also present in the younger generation, but only with women (Table 3). Younger women (closer to 19 years) were more likely to be social media users, with a predicted probability of 97% compared to the predicted probability of 72% for 56-year-old women (see Supplementary Table S5 in Appendix). Interactions showed that the association between age and social media use differed depending on gender and generation, with the age-gender interaction being significant only in the younger generation (*ß* = 0.06, *p* < 0.05).

**TABLE 2:** Predictors of social media use in older Finnish adults (born 1945–1950) (*n* = 2,663). β-coefficients and (standard errors).

Older generation (68–73 years)	All	Women	Men
Age	−0.076**	−0.062	−0.094*
	(0.027)	(0.035)	(0.042)
Gender (man)	0.336***		
	(0.097)		
Education (Elementary school)			
Upper secondary/Vocational	0.193	0.017	0.456**
	(0.107)	(0.136)	(0.176)
College/Lower university degree	0.302	0.163	0.450
	(0.170)	(0.216)	(0.284)
Higher academic degree	0.577***	0.621**	0.589**
	(0.157)	(0.219)	(0.239)
Economic situation	−0.015	−0.004	−0.047
	(0.064)	(0.083)	(0.103)
Marital status (Married/Cohabiting)			
Unmarried	−0.151*	−0.177	−0.310
	(0.210)	(0.260)	(0.391)
Divorced	0.206	0.056	0.559*
	(0.142)	(0.167)	(0.275)
Widowed	0.133	0.028	0.427
	(0.150)	(0.176)	(0.297)
Health	0.233***	0.333***	0.104
	(0.063)	(0.084)	(0.099)
Number of close friends	0.017	0.050*	−0.000
	(0.013)	(0.021)	(0.017)
Number of close relatives	0.015	0.012	0.019
	(0.008)	(0.010)	(0.014)
Number of children	0.46***	0.100*	0.205***
	(0.034)	(0.045)	(0.051)
Contact frequency with closest friend	0.140***	0.186^***^	0.080
	(0.040)	(0.052)	(0.064)
Contact frequency with closest relative	0.010	0.050	−0.026
	(0.044)	(0.060)	(0.067)
_cons	2.933	1.787	4.782
	(1.934)	(2.544)	(3.044)
*n*	2,239	1,316	923
pseudo R^2^	0.043	0.046	0.043

*p < 0.05,**p < 0.01,***p < 0.001.

**TABLE 3:** Predictors of social media use in young and middle-aged Finnish adults (born 1962–1999) (*n* = 1945). β-coefficients and (standard errors).

Younger generation (19–56 years)	All	Women	Men
Age	−0.043***	−0.075***	−0.011
	(0.012)	(0.018)	(0.017)
Gender (man)	0.963***		
	(0.097)		
Education (Elementary school)			
Upper secondary/Vocational	0.681*	0.971	0.340
	(0.341)	(0.516)	(0.452)
College/Lower university degree	1.234***	1.352*	1.022*
	(0.360)	(0.540)	(0.484)
Higher academic degree	0.938**	1.005	0.713
	(0.358)	(0.539)	(0.480)
Economic situation	0.173	0.206	0.213
	(0.093)	(0.136)	(0.132)
Marital status (Married/Cohabiting)			
Unmarried	0.153	−0.100	0.462
	(0.193)	(0.267)	(0.375)
Divorced/Widowed	0.336	0.809*	−0.092
	(0.241)	(0.377)	(0.337)
Health	0.012	0.020	0.025
	(0.088)	(0.124)	(0.127)
Number of friends	0.011	0.044	−0.006
	(0.021)	(0.037)	(0.025)
Number of close relatives	−0.016	−0.023	−0.012
	(0.017)	(0.024)	(0.024)
Number of children	0.126*	0.150*	0.100
	(0.049)	(0.070)	(0.069)
Contact frequency with closest friend	0.208***	0.325***	0.111
	(0.053)	(0.080)	(0.071)
Contact frequency with closest relative	0.026	0.145	−0.067
	(0.067)	(0.096)	(0.094)
_cons	3.118	1.807	5.327
	(1.925)	(2.540)	(3.012)
*n*	2,239	1,316	923
pseudo *R* ^2^	0.037	0.043	0.030

*p < 0.05,**p < 0.01,***p < 0.001.

Women were more likely social media users in both generations. In the older generation, women’s likelihood of being social media users was 46% and men’s likelihood was 38%. In the younger generation, women’s likelihood of being social media users was 86% and men’s was 71% (see Supplementary Tables S1, S4 in the Appendix). Gender was among the clearest predictors of social media use in both generations; however, the association was weaker in the older generation (*ß* = 0.70, *p* < 0.001).

Education was also a significant predictor of social media use. Those with higher education were more likely to be social media users than those with less education. Predicted probabilities in the older generation range from the lowest category of education at 38–51% in those with higher education (see Supplementary Table S1 in the Appendix). In the younger generation, the lowest and highest predicted probabilities were 65% for the least educated and 85% for those with college-level or lower university degrees (see Supplementary Table S4 in the Appendix).

Health predicted social media use only among older generation women (Table 2). Those who rated their health as very good were twice as likely to be social media users than those who rated their health as very poor (59 and 28%, respectively; see Supplementary Table S2 in the Appendix).

Of the categories representing marital status, only divorce was related to social media use (Tables 2, 3). A three-way interaction showed that the effect of divorce differed depending on gender and generation (*ß* = 2.29, *p* < 0.05), with older divorced men and younger divorced women being more likely social media users. In the older generation, the predicted probability of being a social media user for a divorced man was 49%, compared to 36% for his married counterpart (see Supplementary Table S3 in the Appendix). Conversely, in the younger generation, the predicted probability of being a social media user for a divorced woman was 93%, compared to 86% for her married counterpart (see Supplementary Table S5 in the Appendix).

Of the characteristics of the respondents’ social lives, the number of children was positively associated with social media use in both men and women of the older generation (Table 2) and in the women of the younger generation (Table 3). For example, the predicted probability of being a social media user was 35% for a childless respondent from the older generation, compared to 39% for those with one child and 42% for those with two children (see Supplementary Table S1 in the Appendix). In the younger generation, the predicted probability of being a user rose from 83% for a childless woman to 85% for a woman with one child and 87% for a woman with two children (see Supplementary Table S5 in the Appendix).

The number of friends was also positively associated with social media use among women of the older generation (Table 2). The effect was modest, with the predicted probabilities of being a social media user increasing from 41% for those older women who reported having zero friends to 42% for those who had one friend and 43% for those who had two friends. The ceiling value for the number of friends was set at 20, the value at which the predicted probability that a women of the older generation was a social media user was 64%. The most common value for the number of friends among women of the older generation, however, was four, with a predicted probability of social media use of 45% (see Supplementary Table S2 in the Appendix).

Contact frequency with closest friends was associated with social media use in women of both generations (Tables 2, 3); however, the association was stronger in the women of the younger generation (*ß* = 0.21, *p* < 0.05). In the older generation, the women who reported having no contact with their closest friend (including those who did not have a close friend) had a predicted probability of 35% of being social media users. The predicted probabilities increased as contact frequency increased. For example, the predicted probability was 40% for the response option “less than once a month” but 44% for the most common response option “1–3 times a month” (see Supplementary Table S2 in the Appendix). In the younger generation of women, the equivalent probabilities were 78 and 83%; however, the most common value for frequency of contact with closest friend was “Daily or several times per week,” with a predicted probability of social media use of 90% (see Supplementary Table S5 in Appendix).

Contact frequency with relatives and the number of close relatives were not associated with social media use.

**FIGURE 1:**
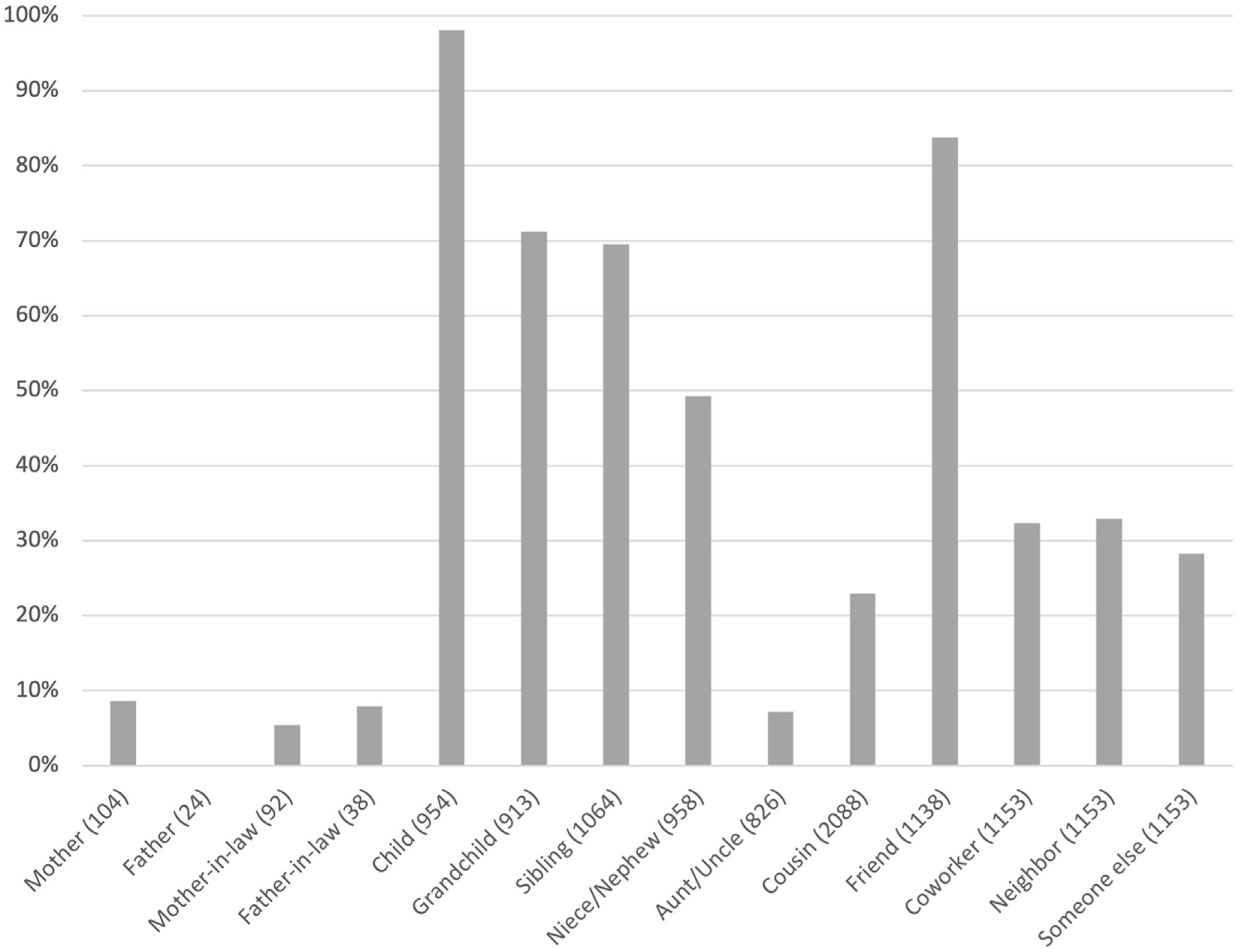
Percentage of respondents in the older generation (aged 68–73) connected with kin and non-kin via social media. In the case of relatives, (*n*) indicates the number of respondents with the given relative.

**FIGURE 2:**
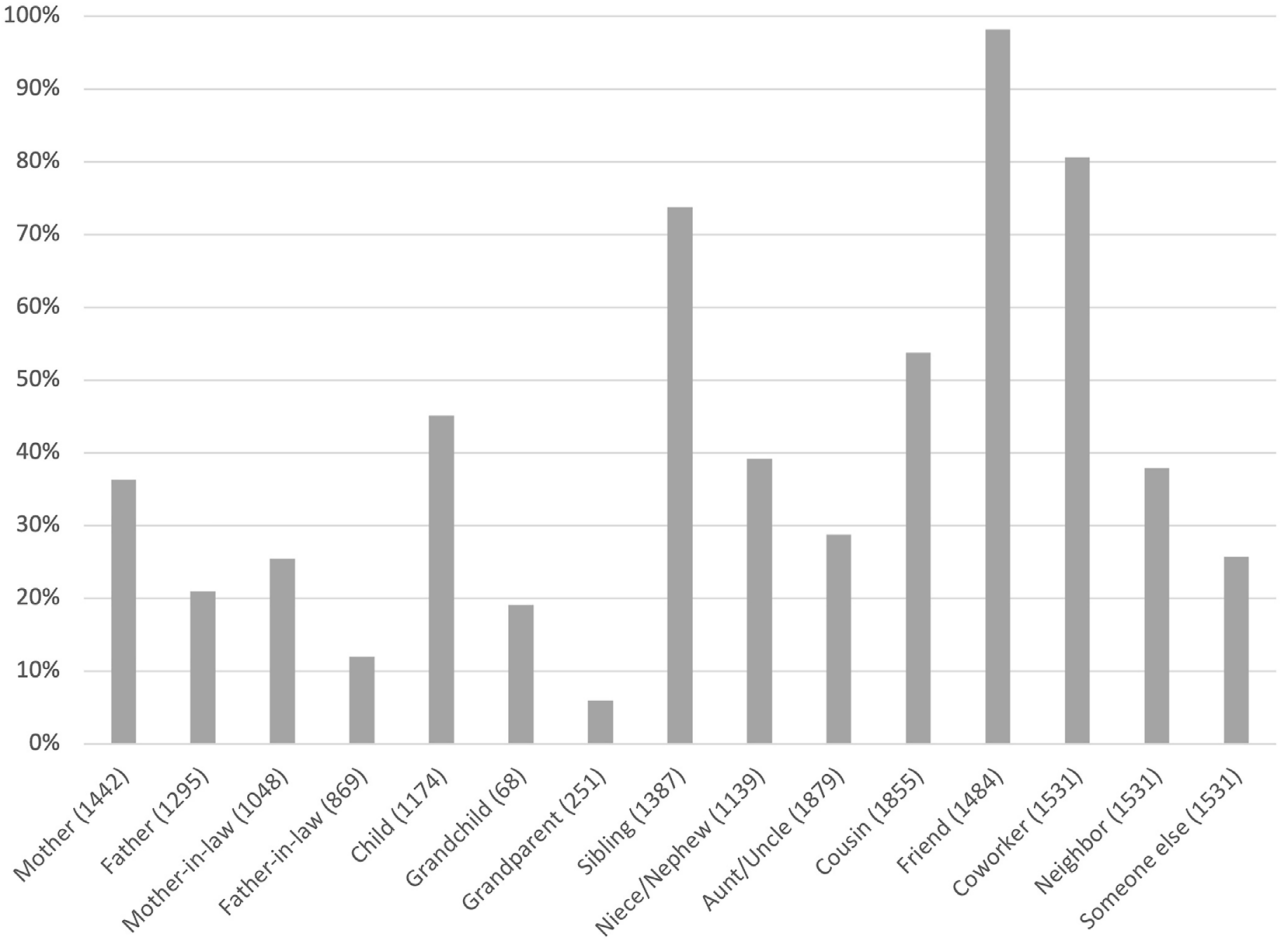
Percentage of respondents in the younger generation (aged 19–56) connected with kin and non-kin via social media. In the case of relatives, (*n*) indicates the number of respondents with the given relative.

Figures 1, 2 show with whom social media users had contact via social media. Respondents of the older generation most frequently reported using social media to interact with their children (98%). The second most frequent category was friends (84%), and the third and fourth most frequent categories were grandchildren (71%) and siblings (70%).

Regarding respondents of the younger generation, they most frequently reported using social media to interact with friends (98%) and coworkers (81%). The younger generation clearly interacted less with their parents via social media (37% with mothers and 21% with fathers) than their parents’ generation did with their adult children (98%), which is at least partially explained by the lower rates of social media use among the older adults. With siblings, however, the younger generation interacted approximately as frequently (74%) as did their parents’ generation (70%).

The relatively low percentage of younger generation respondents who interacted with their children on social media, that is 45%, can partially be explained by the fact that most had children who were too young to be social media users. However, even when excluding respondents with under 18-year-old children, the percentage of those who interacted with their children via social media remained clearly below that of the older generation (61 and 98%, respectively).

Last, we tested whether social media use in older adults was predicted by the social media use of their adult children. To study this question, we used data that included actual parent-child dyads from the same families (*n* = 1,003). We found that the social media use of an adult child was a significant predictor of the social media use of their parents. After controlling for age and gender, 43% of older adults whose children were social media users were also social media users, whereas the percentage was only 32% for those older adults whose children were not social media users (*p* < 0.05).

## Discussion

This study examined social media use in two generations of Finnish adults. Social media use was almost twice as prevalent in the younger generation (80%) than it was in the older generation (42%). When comparing the generations, our results show that there are differences in the predictors of social media use. As pointed out by Yu et al. (2016), predictors of social media use may be life-stage specific. In our representative sample of Finnish adults, we found that generation-varying determinants include gender, age, divorce, and frequency of contact with friends. Next, we discuss the predictors of social media use in light of previous evidence.

As established in many previous studies, women, younger individuals, and the more educated are more typical social media users (Bell et al., 2013; Richter et al., 2013; Hutto et al., 2015; Vošner et al., 2016; Yu et al., 2016; Anderson and Perrin, 2017; Newman et al., 2019). The present study also showed these associations in both generations; however, both the gender bias and the effect of age were less pronounced in the older generation.

Predictors of social media use that were specific to the older generation included better health in women. This finding contradicts previous studies that did not find an association between health and social media use (Yu et al., 2016; Yu, 2020). To account for these incongruent results, we propose that the association may become observable only when considering women separately and within a narrow age range, such as the sample used here (i.e., 68–73 years). Yu and colleagues’ studies, in comparison, considered wider age ranges, from 50 to 60 and beyond, and they did not examine genders separately.

Predictors that affected younger and older generations differently included marital status. Divorced women (incl. widows) in the younger generation and divorced men in the older generation formed pockets of more frequent social media users. In contrast to our findings, Yu et al. (2016) did not find divorced individuals over the age of 50 to be more likely users of social media. However, they did find that widows form a group of more frequent users, concluding that social media can play a compensatory role for those who have experienced loss.

As regards the characteristics of the respondents’ social lives, in both generations, the more children the respondents had, the more likely they were to be social media users; however, in the younger generation, the association was observable only in women. These findings indicate that children may play a significant role in the social media adoption of their parents. Possible mechanisms include active encouragement (Bell et al., 2013; Jung et al., 2017; Aarts, 2018; Yu, 2020) and technical assistance (Aarts, 2018; Hänninen et al., 2021). Moreover, for many individuals, social media provides new ways to increase involvement in the lives of children and grandchildren, and this may drive social media adoption in parents and grandparents. Many studies have found this to be the primary self-reported reason for using social media in older adults (e.g., Zickuhr and Madden, 2012; Nef et al., 2013; Aarts, 2018; Newman et al., 2019; Yu, 2020).

Another characteristic of social life associated with social media use was contact frequency with close friends. This was significant in women of both generations. Similarly, previous studies have shown that older adults who frequently meet friends or family offline are more likely to be social media users (e.g., Richter et al., 2013). Our findings are in line with this previous finding but only when considering women and their contact frequency with their closest friend. In contrast, contact frequency with closest relatives was not associated with social media use, and men showed no associations for either friends or relatives.

In women of the older generation, an additional, albeit negligible, association was observed between the number of friends and social media use. These results tentatively support the notion that offline social networks can stimulate social media use, at least in women. Unlike our results, previous findings do not show that strong ties (i.e., to close friends and family) make a difference for social media use, indicating instead that social media use is positively associated with the number of weak ties (i.e., to acquaintances and non-intimate friends) (Yu, 2020). As our results do not indicate that the number of close relatives is associated with social media use, we do not find further evidence for the association between strong-tie network size and social media use.

Looking at the distributions of various kin and non-kin with whom respondents were in contact via social media, we find that the younger generation most often had friends and coworkers as social media contacts, whereas the older generation most often had their children as contacts. Our results regarding the prevalence and purpose of social media use are in line with United States trends (Hutto et al., 2015; Zickuhr and Madden, 2012; Vogels, 2019). Thus, it seems that in affluent Western societies, social media use is becoming increasingly widespread among older adults. Our results also add to the evidence that, with age, the focus of social media use shifts from non-kin to family (see also Zickuhr and Madden, 2012; Hutto et al., 2015), indicating that family members may be a major determinant of social media use among older adults.

Finally, we investigated whether social media use in a parent, representing the older generation, is predicted by social media use in their adult children, representing the younger generation. The Gentrans survey identifies family lineages and, therefore, enables the combination of results from related individuals from older and younger generations. The results indicate that the social media use of an adult child was a significant predictor of the social media use of a parent.

Taken together, the findings regarding the characteristics of both younger and older adults’ social lives and their associations with social media use seem to indicate that social media play a complementary role in their present form and with their present functions (see also Yu et al., 2016). This means that those with existing social capital, either in the form of an active social life or a number of strong-tie relationships (including children), are more likely to be social media users. Although these aspects of social life influence social media use in both generations, their influence becomes more pronounced in older adults.

Conversely, the widowed or childless have not adopted social media, and neither have those who have been deprived of friends or who have health issues. Therefore, social media do not appear to be used in a compensatory manner, that is, to mitigate a lack of social contacts. The architecture of social media may be better suited for complementing existing close relationships than for forming new ones. There may be significant potential here for development in terms of both digital solutions and geriatric care.

Although the present study has several strengths, it is not without limitations. In the Gentrans surveys, social media was defined as social networking sites (i.e., Facebook, Instagram, Twitter, and Snapchat). However, social media has increasingly diverged into two different types of platforms: messaging and networking. These two factors may have different implications for family communication and the well-being derived from such communication (Yu, 2020). In addition, the contact frequency variable used here comprised all methods of communication, including contact *via* social media. Therefore, the result regarding frequency of contact can be interpreted in terms of opposite causality, that is, that social media increases contact frequency. Finally, in the analysis, we controlled for several potential confounding factors, but all such factors are difficult if not impossible to account for, and so, we cannot claim that the associations found are causal. We also acknowledge that opposite and bidirectional causal pathways are possible and could change the interpretations of the results. For example, in predicting social media use of an older adult by the social media use of their child, it is important to keep in mind that the results also support an inverted causal interpretation.

To conclude, uneven technology adoption among an aging population forms a digital divide that may further exacerbate inequalities. In other words, while one section of the population reaps the benefits of modern technology, including the possibility of strengthening informal support networks, another section of the population lacks both social support and the means to get online. An increasingly digital society may face deepening divides if the technology adoption of older adults depends on whether they have social networks that can help them to get connected.

## Data Availability

The data analyzed in this study is subject to the following licenses/restrictions: Gentrans surveys will be available for scholars *via* Finnish Social Science Data Archive after embargo. Requests to access these datasets should be directed to https://blogs.helsinki.fi/gentrans/.

## References

[B1] Aarts S. (2018). Social media and Loneliness Among Community-Dwelling Older Adults. Int. J. Geriatr. Psychiatry 33, 554–555. 10.1002/gps.4769 29424110

[B2] Anderson M. Perrin A. (2017). Tech Adoption Climbs Among Older Adults. Washington: Pew Research Center. Available at: https://www.pewresearch.org/internet/2017/05/17/tech-adoption-climbs-among-older-adults/ .

[B3] Baumer E. P. S. (2018). Socioeconomic Inequalities in the Non Use of Facebook, in Paper presented at CHI 2018, 1–14. 10.1145/3173574.3174190

[B4] Bell C. Fausset C. Farmer S. Nguyen J. Harley L. Fain W. B. (2013). Examining Social media Use Among Older Adults. In Proceedings of the 24th ACM conference on Hypertext and Social Media (HT ’13). New York, NY: Association for Computing Machinery, 158–163. 10.1145/2481492.2481509

[B5] Büchi M. Just N. Latzer M. (2016). Modeling the Second-Level Digital divide: A Five-Country Study of Social Differences in Internet useModeling the Second-Level Digital divide: A Five-Country Study of Social Differences in Internet Use. New Media & SocietyNew Media Soc 18, 2703–2722. 10.1177/1461444815604154

[B6] Chan M. (2015). Multimodal Connectedness and Quality of Life: Examining the Influences of Technology Adoption and Interpersonal Communication on Well-Being across the Life Span. J Comput-Mediat Comm 20, 3–18. 10.1111/jcc4.12089

[B7] Chen Y. R. Schulz P. J. (2016). The Effect of Information Communication Technology Interventions on Reducing Social Isolation in the Elderly: A Systematic Review. J. Med. Internet Res. 18, e18. 10.2196/jmir.4596 26822073 PMC4751336

[B8] Cornejo R. Tentori M. Favela J. (2013). Enriching In-Person Encounters through Social media: A Study on Family Connectedness for the Elderly. International Journal of Human-Computer Studies 71, 889–899. 10.1016/j.ijhcs.2013.04.001

[B9] Danielsbacka M. Tanskanen A. O. (2016). The Association between Grandparental Investment and Grandparents' Happiness in Finland. Pers Relationship 23, 787–800. 10.1111/pere.12160

[B10] Friemel T. N. (2016). The Digital divide Has Grown Old: Determinants of a Digital divide Among seniorsThe Digital divide Has Grown Old: Determinants of a Digital divide Among Seniors. New Media & SocietyNew Media Soc 18, 313–331. 10.1177/1461444814538648

[B11] Gaia A. Sala E. Cerati G. (2020). Social Networking Sites Use and Life Satisfaction. A Quantitative Study on Older People Living in EuropeSocial Networking Sites Use and Life Satisfaction. A Quantitative Study on Older People Living in Europe. European Societies 23, 98–118. 10.1080/14616696.2020.1762910

[B12] Hanifi R. (2019). Vapaa-aikatutkimus. Tilastokeskus, Statistics Finland: Research on Leisure Time.

[B13] Hänninen R. Taipale S. Luostari R. (2021). Exploring Heterogeneous ICT Use Among Older Adults: The Warm Experts' perspectiveExploring Heterogeneous ICT Use Among Older Adults: The Warm Experts’ Perspective. New Media & SocietyNew Media Soc 23, 1584–1601. 10.1177/1461444820917353

[B14] Hutto C. J. Bell C. Farmer S. Fausset C. Harley L. Nguyen J. (2015). Social media Gerontology: Understanding Social media Usage Among Older Adults. Web 13, 69–87. 10.3233/WEB-150310

[B15] Jung E. H. Walden J. Johnson A. C. Sundar S. S. (2017). Social Networking in the Aging Context: Why Older Adults Use or Avoid Facebook. Telematics and Informatics 34, 1071–1080. 10.1016/j.tele.2017.04.015

[B16] Lüders M. Brandtzæg P. B. (2017). 'My Children Tell Me It's So Simple': A Mixed-Methods Approach to Understand Older Non-users' Perceptions of Social Networking Sites. New Media & SocietyNew Media Soc 19, 181–198. 10.1177/1461444814554064

[B17] Luijkx K. Peek S. Wouters E. (2015). "Grandma, You Should Do It--It's Cool" Older Adults and the Role of Family Members in Their Acceptance of Technology. Int. J. Environ. Res. Public Health 12, 15470–15485. 10.3390/ijerph121214999 26690188 PMC4690935

[B18] Nef T. Ganea R. L. Müri R. M. Mosimann U. P. (2013). Social Networking Sites and Older Users - A Systematic Review. Int. Psychogeriatr. 25, 1041–1053. 10.1017/S1041610213000355 23552297

[B19] Newman L. Stoner C. Spector A. (2019). Social Networking Sites and the Experience of Older Adult Users: A Systematic Review. Ageing and Society 41, 377–402. 10.1017/S0144686X19001144

[B20] Nguyen M. H. Hunsaker A. Hargittai E. (2020). Older Adults' Online Social Engagement and Social Capital: The Moderating Role of Internet Skills. Information, Communication & Society 1, 1–17. 10.1080/1369118X.2020.1804980

[B21] Richter D. Bannier S. Glott R. Marquard M. Schwarze T. (2013). Are Internet and Social Network Usage Associated with Wellbeing and Social Inclusion of Seniors? - The Third Age Online Survey on Digital media Use in Three European Countries, Lecture Notes in Computer Science. Berlin Heidelberg: Springer, 211–220. 10.1007/978-3-642-39191-0_24

[B22] Simons M. Reijnders J. Peeters S. Janssens M. Lataster J. Jacobs N. (2021). Social Network Sites as a Means to Support Personal Social Capital and Well-Being in Older Age: An Association Study. Computers in Human Behavior Reports 3, 100067. 10.1016/j.chbr.2021.100067

[B23] Statistics Finland (2020). Väestön Tieto- Ja Viestintätekniikan Käyttö [Information and Communication Technology Adoption in the Population]. ISSN=2341–8699. Helsinki: Tilastokeskus. Available at: https://www.stat.fi/til/sutivi/2020/sutivi_2020_2020-11-10_tie_001_fi.html .

[B24] van Deursen A. J. A. M. Helsper E. J. (2015). The Third-Level Digital divide: Who Benefits Most from Being Online? Studies in Media and Communications 10, 29–52. 10.1108/S2050-206020150000010002

[B25] Vogels E. A. (2019). Millennials Stand Out for Their Technology Use, but Older Generations Also Embrace Digital Life. Washington: Pew Research Center. Available at: https://pewrsr.ch/2A3kD6X .

[B26] Vošner H. B. Bobek S. Kokol P. Krečič M. J. (2016). Attitudes of Active Older Internet Users towards Online Social Networking. Computers in Human Behavior 55, 230–241. 10.1016/j.chb.2015.09.014

[B27] Yu R. P. Ellison N. B. McCammon R. J. Langa K. M. (2016). Mapping the Two Levels of Digital divide: Internet Access and Social Network Site Adoption Among Older Adults in the USA. Information, Communication & Society 19, 1445–1464. 10.1080/1369118X.2015.1109695

[B28] Yu R. P. (2020). Use of Messaging Apps and Social Network Sites Among Older Adults: A Mixed-Method Study. Int. J. Commun. 14, 4453–4473. Available at: http://ijoc.org .

[B29] Zickuhr K. Madden M. (2012). Older Adults and Internet Use. Washington: Pew Research Center. Available at: http://www.pewresearch.org/internet/2012/06/06/main-report-15 .

